# The predominant species of nonstructural protein 4B in hepatitis C virus-replicating cells is not palmitoylated

**DOI:** 10.1099/vir.0.000111

**Published:** 2015-07

**Authors:** David Paul, Ralf Bartenschlager, Christopher McCormick

**Affiliations:** ^1^​ Department of Infectious Diseases, Molecular Virology, University of Heidelberg, 69120 Heidelberg, Germany; ^2^​ Faculty of Medicine and Institute for Life Science, University of Southampton, Southampton SO16 6YD, UK

## Abstract

Hepatitis C virus (HCV) represents a significant global health burden. Viral replication is thought to occur in close association with remodelled host cell membranes, with non-structural protein 4B (NS4B) being a key player in this process. NS4B is a poorly characterized integral membrane protein, which has been reported to be palmitoylated at its carboxy-terminal end. In order to extend this observation and to establish a functional role for NS4B palmitoylation, we sought to determine the status of this post-translational modification when the protein was expressed as part of a functional viral replicase. We performed direct metabolic labelling and polyethylene glycol-maleimide palmitoylation reporter assays on NS4B expressed in cells containing subgenomic replicons and infectious viral RNA. In a vaccinia virus-based expression system NS4B palmitoylation was detected in a genotype-dependent manner. However, in spite of the high sensitivity of the methods used, no NS4B palmitoylation was found in physiologically more relevant systems. Thus, NS4B palmitoylation is most likely dispensable for HCV RNA replication.

Hepatitis C virus (HCV) infects an estimated 170 million individuals worldwide, and is responsible for significant liver related morbidity and mortality. As a member of the recently classified hepacivirus genus, it is a positive strand enveloped RNA virus encoding both structural and non-structural (NS) proteins within a single open reading frame, translation of which is driven by an internal ribosome entry site. Replication of the HCV genome requires remodelling of host-cell derived endoplasmic reticulum (ER) membranes to form the viral replication factory (vRF), a membranous compartment that sequesters viral and host cell proteins necessary for RNA synthesis and protects replicative intermediates from antiviral host activity (reviewed by Paul *et al.*, 2014). A key viral protein involved in membrane remodelling events is NS4B (Egger *et al.*, 2002). While the full structure of NS4B has yet to be solved, the protein is believed to possess two amino-terminal amphipathic helices, four central trans-membrane domains and two α-helices in the carboxy-terminal domain (reviewed by Gouttenoire *et al.*, 2010a). NS4B self-interacts via multiple determinants, which is required for its function in vRF biogenesis (Gouttenoire *et al.*, 2010b; Paul *et al.*, 2011). Expressed alone, NS4B induces the formation of single membrane vesicles within cells, but needs to be expressed in the context of the NS3-5B replicase to recapitulate the same membrane changes found in infected cells (Romero-Brey *et al.*, 2012). One feature of NS4B that may be important for its function is palmitoylation, a post-translational modification mapped to two cysteine residues found at the C-terminal end of the protein (Yu *et al.*, 2006) (Fig. 1a).

**Fig. 1.**
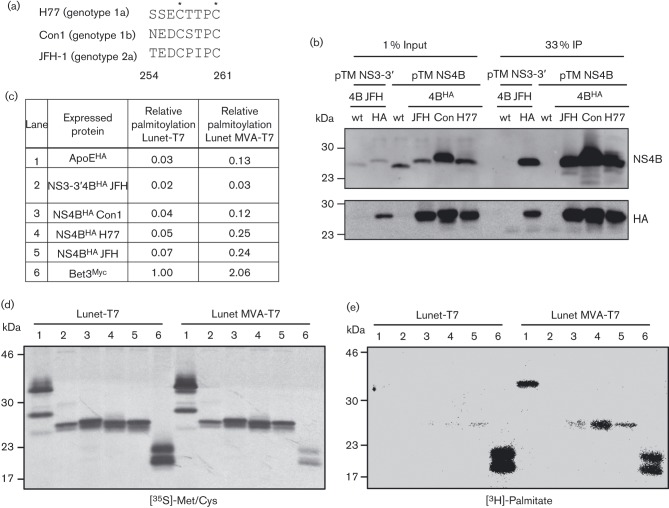
The non-palmitoylated status of NS4B is altered upon MVA-T7-driven protein expression. Shown is a sequence alignment of the carboxy-terminal region of NS4B (amino acids 254–261) for the three HCV isolates used in this study, with asterisks indicating cysteine residues previously reported to be palmitoylated (a). Huh7-Lunet/T7 cells transfected with pTM expression plasmids as indicated on the top were harvested 24 h after transfection and NS4B was immunocaptured with HA-beads. Western blot analysis with mono-specific antibodies indicated on the right is shown in (b). After transfection of cells with pTM and control expression plasmids as indicated in (c), metabolic labelling with either [^3^H]-palmitate or [^35^S]-Met/Cys was performed. Proteins were purified by HA-, or Myc-specific IP and after SDS-PAGE the [^35^S]-labelled proteins were visualized by autoradiography (d) and the [^3^H]-signal was detected using a β-imager (e). A total of 33 % of the IP fraction was loaded in each lane and molecular mass standards are indicated on the left of each blot. (c) Relative palmitoylation of the respective protein was calculated as a ratio of [^3^H]/[^35^S] signal normalized for the sum of Met/Cys residues in the given protein and assuming a single palmitoylation site. Values that are normalized to the Bet3 positive control expressed in Lunet-T7 cells are shown.

The aim of this study was to extend the original observations regarding NS4B palmitoylation by quantifying its occurrence when the protein was expressed in a context that supports HCV RNA replication, thus establishing its likely importance in replication complex formation. In the first instance, we expressed NS4B from two different T7-driven protein expression systems using constructs encoding NS4B with a haemagglutinin (HA)-epitope tag inserted after residue 38, either in the context of a JFH-1 NS3-5B polyprotein, or as a single protein derived from isolates JFH1, Con1 and H77. Analysis of cell lysates after anti-HA pull down confirmed that the tag allowed specific purification of NS4B in all cases (Fig. 1b). Next we sought to determine the NS4B palmitoylation status by metabolic labelling with tritiated palmitate and compared a cell line that stably overexpressed T7 RNA polymerase (Lunet-T7) to the transient T7 delivery by infection of naive Lunet cells with a modified vaccinia virus (MVA-T7), as performed previously (Yu *et al.*, 2006). Expression of Bet3 served as a positive control for palmitoylation, while expression of HA-tagged apolipoprotein E (ApoE^HA^) was used to control for non-specific background labelling. A total of 3.5×10^5^ cells per well were seeded into 6-well plates and transfected with expression vectors 24 h later. In the case of vaccinia virus-mediated T7 expression, cells were infected with MVA-T7 90 min prior to transfection of plasmids. Sixty eight hours post seeding, cells were starved for 1 h in medium with delipidated FCS and subsequently labelled with 80 µCi ml^−1^ [9,10-^3^H(*N*)]-palmitate for 3 h. In parallel we labelled total proteins, supplying cells with 20 µCi ml^−1^ [^35^S]-Met/Cys. Cells were lysed in RIPA buffer (50 mM Tris pH 7.5, 150 mM NaCl, 1 % NP-40, 0.5 % deoxycholate, 0.1 % SDS, 1× protease inhibitors) and immuno-purified using anti-HA or anti-Myc agarose beads for NS4B^HA^, ApoE^HA^ and Bet3^Myc^, respectively. After elution of proteins in non-reducing sample buffer they were separated by SDS-PAGE, blotted onto PVDF membranes and analysed with a β Imager 2000 (Biospace Lab) to detect the ^3^H radioactive signal. Detection of ^35^S signals by autoradiography confirmed successful immunoprecipitation (IP) of all proteins and revealed comparable expression and recovery of NS4B in Lunet-T7 cells and MVA-T7 infected Lunet cells (Fig. 1d). Furthermore, in both experimental conditions we detected palmitoylation of Bet3 as reported earlier (Turnbull *et al.*, 2005) (Fig. 1e). Intriguingly, while palmitoylation of NS4B was not detectable in Lunet-T7 cells, we observed robust ^3^H radioactive signals upon MVA-T7-mediated NS4B expression. Importantly, the negative control ApoE was also found to be profoundly palmitoylated in the latter condition, suggesting a strong influence on protein palmitoylation by MVA infection. Quantification of these signals further allowed us to estimate the degree of NS4B palmitoylation (^3^H band intensity) by normalizing to the total amount of immunopurified protein (^35^S band intensity divided by the number of Met+Cys residues) (Fig. 1c). This analysis revealed a substantial increase in palmitoylation of NS4B, when expressed as a single protein, but also of the negative control protein ApoE, in the MVA-T7 experimental condition. Thus, at least under given experimental conditions, vaccinia virus stimulated non-specific palmitoylation of proteins.

To avoid further use of MVA-T7-driven protein expression we adopted a more physiologically relevant system, performing metabolic labelling with tritiated palmitate on cells that stably replicate a subgenomic JFH RNA, encoding an HA-epitope in the N-terminal region of NS4B (Paul *et al.*, 2013). The ER-resident protein calnexin (CANX) served as an additional positive control for palmitoylation (Lakkaraju *et al.*, 2012). The [^3^H]-palmitate labelling and subsequent immunocapture of proteins was performed as described above. Western blot analysis with mono-specific antibodies revealed that all proteins were specifically enriched after IP (Fig. 2a). However, no NS4B palmitoylation was detected in contrast to robust signals for the positive controls CANX and Bet3 (Fig. 2b).

**Fig. 2.**
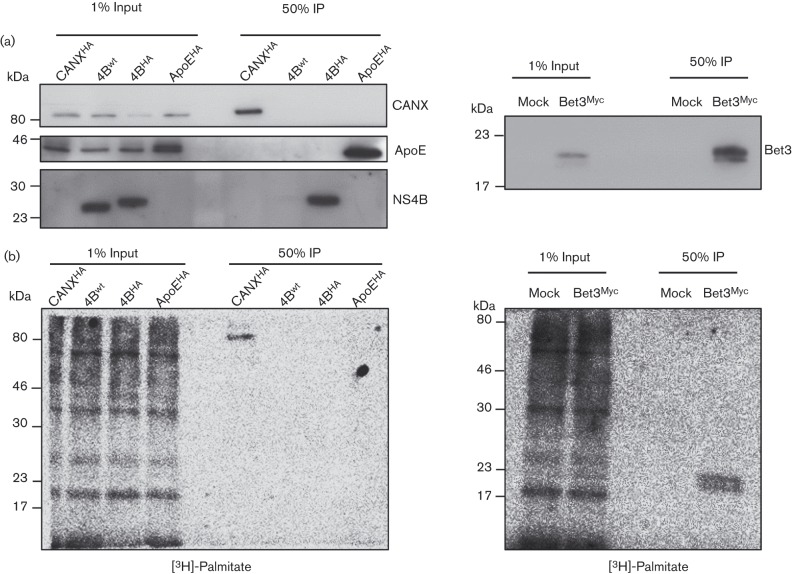
Absence of detectable palmitoylation of NS4B purified from active HCV replication complexes. Huh7-Lunet cells overexpressing calnexin-HA (CANX^HA^), or containing a JFH-1 wild-type replicon (NS4B^wt^) or a JFH-1 replicon encoding HA-tagged NS4B (NA4B^HA^), or transfected with expression vectors encoding HA-tagged apolipoprotein E (ApoE^HA^) or the Myc-tagged Bet3 positive control protein (Bet3^Myc^), were metabolically labelled with [^3^H]-palmitate followed by HA- or Myc-specific IP. Western blot analyses of unlabelled control samples that were processed in parallel are shown in (a). Conjugation of radioactive [^3^H]-palmitate to proteins was revealed using a β-imager and is depicted in (b). Molecular mass standards are indicated on the left of each blot.

In addition to our attempts to directly visualize palmitoylation by metabolic labelling, we assessed this modification using an established chemical-based procedure (Fig. 3) (Roth *et al.*, 2006). In this assay, *N*-ethylmaleimide (NEM) was used to irreversibly block all free thiol groups on cysteine residues. Hydroxylamine was subsequently used to specifically reduce the thioester bonds linking cysteine residues to palmitic acid, making them susceptible to modification with PEG-maleimide, detectable through a reduction in electrophoretic mobility of PEG-modified proteins (Fig. 3a).

**Fig. 3.**
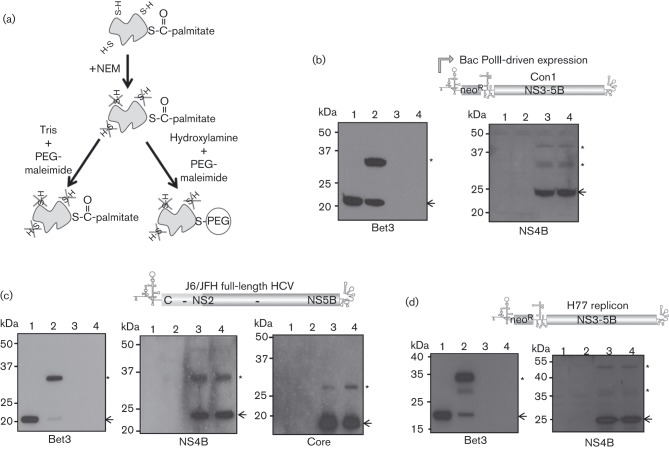
Assessing palmitoylation of NS4B in HCV replication complexes by maleimide-based chemistry. The illustration (a) shows the step-wise chemical reaction these NS4B proteins were subjected to. In this assay, free thiol groups are first blocked by *N*-ethylmaleimide (NEM). Enhanced conjugation to PEG-maleimide subsequent to treatment with hydroxylamine versus treatment with Tris indicates palmitoylation, visualized as an increase in the ratio of PEG-modified protein to unmodified protein. Western blots show the results on NS4B palmitoylation from cells expressing genotype 1b (b), 2a (c) and 1a (d) HCV polyproteins. Polyprotein expression was achieved using an RNA polymerase II-based baculovirus delivery system to introduce the FK5.1neo transcript into HepG2 cells (b), by transfection of an infectious full-length genotype 2a transcript (containing a chimeric JFH-1[nt1–77]/J6[nt 78–341] 5′UTR and encoding a J6/JFH-1 polyprotein equivalent to Jc1) (Pietschmann *et al.*, 2006) into Huh7.5 cells (c), or use of a stable Huh7.5-derived cell line carrying the H77 H/SG-Neo(L+I) replicon (d). HepG2 (b) or Huh7.5 (c, d) cells transfected with the plasmid pBet3-myc were used as a positive control in each assay. In the experimental group transfected with the genotype 2a infectious HCV transcript, assessment of core palmitoylation was included as a further control (c). Lanes 1 and 2 represent the pBet3-myc transfected cell lysates, lanes 3 and 4 the HCV lysates, with proteins having been treated with Tris (lanes 1, 3) or hydroxylamine (lanes 2, 4) prior to incubation with PEG-maleimide. The arrows on the blots indicate the position of unmodified protein and the asterisks indicate the position of PEG-modified protein. Molecular mass standards are indicated on the left of each blot.

To facilitate detection of NS4B, a baculovirus delivery system was initially used to drive high level production of a Con1 subgenomic replicon transcript in the hepatocyte cell line HepG2. A 25 cm^2^ flask seeded 24 h earlier with 7.5×10^5^ cells was transduced for 24 h with 6.25×10^6^ p.f.u. ml^−1^ of BACrep 5.1neo and BACtTA (McCormick *et al.*, 2004), then lysed in 100 µl TES buffer (50 mM Tris pH 7.4, 150 mM NaCl, 5 mM EDTA, 0.5 % TX-100, 0.1 % SDS) containing 10 mM NEM and 2× protease inhibitor. After clarification of the supernatant by centrifugation, SDS was added to a final concentration of 0.5 % (w/v) and the reactions left overnight at 4 °C. Remaining NEM was removed by three rounds of methanol/chloroform precipitation and the protein pellet dissolved in 20 µl PR buffer (4 % SDS, 50 mM Tris pH 7.4, 5 mM EDTA). Half was added to 40 µl 1 M hydroxylamine (pH 8.0), while the remaining half was added to 40 µl 1 M Tris (pH 8.0) and samples were incubated for 1 h at 25 °C. Each reaction was subjected to a further methanol/chloroform precipitation step to recover protein, the pellet resuspended in 20 µl PR buffer and a further 20 µl 10 % (w/v) PEG-maleimide in TES lysis buffer added. After incubation at 37 °C for 1 h, samples were reprecipitated and analysed by Western blot with mono-specific antibodies. No discernible difference could be seen between the ratio of PEG-conjugated and unconjugated NS4B in the hydroxylamine-versus Tris-treated lysates, indicating that the protein was not palmitoylated (Fig. 3b). In contrast, palmitoylation of a control protein, Bet3, was readily detected.

In case the inability to detect NS4B palmitoylation using the PEG-maleimide assay was a consequence of expressing the replicon transcript in a cell line unable to support RNA replication, or was specific to the Con-1 isolate, we decided to extend our analysis. Therefore a similar assay was performed on a Huh7.5 cell line stably carrying the genotype 1a H77 replicon, and on Huh7.5 cells transfected 96 h earlier with an infectious genotype 2a virus genome. In order to enhance specific detection of NS4B we modified the assay such that cells from a confluent 75 cm^2^ flask were initially lysed in 0.8 ml TX-114 lysis buffer (150 mM NaCl, 10 mM Tris pH 7.4, 2 % TX-114, 1 mM EDTA, 2× complete protease inhibitor) and membrane associated proteins were recovered by phase separation prior to the addition of 10 mM NEM in TES lysis buffer. Neither upon HCV genome transfection, nor in the context of replicating H77 subgenomic RNA, were any signs of NS4B palmitoylation detected (Fig. 3c, d), despite clear evidence of palmitoylation of the Bet3 control each time the assay was run. Furthermore core palmitoylation, reported to occur on cysteine 172 (Majeau *et al.*, 2009), was also visualized using this assay (Fig. 3c), albeit at a lower level than observed for Bet3.

In summary, we do not find evidence for palmitoylation of NS4B in any physiologically relevant setting. This varies from the report by Yu and coworkers, who reported palmitoylation of NS4B (Yu *et al.*, 2006). One reason for this discrepancy might be differences in experimental design. Yu and colleagues entirely relied on infection with a recombinant vaccinia virus to mediate T7-driven expression of NS4B as a single protein. However, VACV infection considerably affects intracellular membrane homeostasis (Krijnse Locker *et al.*, 2013) and it is possible that this might promote ‘false-positive’ NS4B palmitoylation. Indeed, our data support the notion that MVA infection stimulates non-specific palmitoylation of NS4B and ApoE (Fig. 1e).

Another difference is the use of chemical procedures to quantify palmitoylation. Both investigations employed maleimide-based chemistry to target cysteine residues for PEGylation. In our study, PEGylation subsequent to hydroxylamine treatment was used to indicate palmitoylation of proteins, but in the study by Yu *et al.* the presence of a palmitoyl group was instead considered to protect the protein from palmitoylation. Follow-up work we have undertaken is consistent with the notion that hydroxylamine specifically reduces thioester bonds without reducing other oxidized forms of Cys. In contrast a ‘mild’ dithiothreitol treatment, used by Yu and colleagues prior to incubating their protein samples with PEG-maleimide, acts as a non-specific reducing agent in our hands (Fig. S1, available in the online Supplementary Material). As a consequence, differences in NS4B PEGylation observed by Yu and colleagues may have reflected the oxidized status of the cysteine residues in NS4B rather than their palmitoylation status.

In theory, a key test to establish whether NS4B palmitoylation was necessary would be to determine whether a modified version of the protein lacking the C-terminal cysteine residues could support replication. Indeed, Yu *et al.* undertook such analysis and concluded that while cysteine 257 was dispensable for replication, cysteine 261 was absolutely necessary (Yu *et al.*, 2006). However, the interpretation of these findings is hampered by the fact that cysteine 261 is the P1 residue of the NS4B-5A cleavage site, a point acknowledged by the authors at the time. A more recent study by us has shown that the rate of cleavage of this boundary is critical for RNA replication (Herod *et al.*, 2012). Given the central role that cysteine at the P1 position has in allowing efficient recognition by the NS3 protease, it is technically challenging to separate effects caused by polyprotein cleavage or possible palmitoylation defects when introducing mutations at this site. Overcoming this hurdle would require an as yet unavailable trans-complementation system that supports HCV RNA replication independently from polyprotein cleavage.
